# Effect of zinc oxide nanoparticle supplementation in Santa Inês ewes during the transition period

**DOI:** 10.1007/s11250-026-05156-w

**Published:** 2026-06-15

**Authors:** Willinton Hernan Pinchao Pinchao, Henrique de Brito Guedes, Carolina Rodriguez Jimenez, Gabriele Rossini Moreira, Murilo Antônio Fernandes, Bruna Gonçalves Santos Costa, Luciana Morita Katiki, Elisabete Aparecida de Nadai Fernandes, Mateus Oliveira Mena, César Cristiano Bassetto, Ana Cláudia Alexandre de Albuquerque, Alessandro Francisco Talamini do Amarante, Helder Louvandini

**Affiliations:** 1 Laboratório de Nutrição Animal, Centro de Energia Nuclear na Agricultura (CENA), Universidade de São Paulo, Piracicaba, SP Brasil; 2 Instituto de Biociências, Departamento de Biodiversidade e Bioestatística, Universidade Estadual Paulista (UNESP), Botucatu, SP Brasil; 3 Faculdade de Ciências Agrarias e Veterinária, Departamento de Patologia, Reprodução e Saúde única, Universidade Estadual Paulista (UNESP), Jaboticabal, SP Brasil; 4 Laboratório de Radioisótopos, Centro de Energia Nuclear na Agricultura (CENA), Universidade de São Paulo, Piracicaba, SP Brasil; 5 Instituto de Zootecnia (IZ), Nova Odessa, SP Brasil; 6 Center for Nuclear Energy in Agriculture, Laboratory of Animal Nutrition, Avenida Centenário 303, Piracicaba, São Paulo, 13416-000 Brazil

**Keywords:** Antioxidant, Ewe, Immunology, Nanotechnology, Trace element

## Abstract

Zinc oxide nanoparticles (N-ZnO) represent a promising alternative for zinc (Zn) supplementation, since their reduced size provides greater bioavailability and better integration into the body. Furthermore, this characteristic becomes particularly relevant during periods of increased metabolic stress, such as the transition phase. Thus, the objective was to evaluate the effects of N-ZnO supplementation in Santa Inês sheep during the peripartum period. Thirty-three ewes with an average body weight of 43.3 ± 2.05 kg were, distributed into three experimental groups: control group (CON), without supplementation, receiving only *Cynodon* spp. hay; and groups supplemented with 300 mg/animal/day of N-ZnO (40 nm) and zinc oxide microparticles (M-ZnO). The supplementation was administered orally in capsules daily from late pregnancy until weaning. The results showed no significant differences (*P* > 0.05) in body weight, egg count per gram of feces (EPG), and in most of the hematological, biochemical, and immunological parameters evaluated. However, differences (*P* < 0.05) were observed in monocyte counts and albumin levels, with higher means in the N-ZnO group, showing differences compared with the M-ZnO group, while the CON group maintained intermediate values. The N-ZnO group showed differences compared to the M-ZnO group, while the CON group maintained intermediate values. Regarding antioxidant activity, a significant effect (*P* < 0.05) was observed on the enzymes Superoxidase dismutase (SOD), Glutathione peroxidase (GPx), Catalase (Cat), and on the total antioxidant status (ORAC) of the sheep supplemented with N-ZnO. It is concluded that N-ZnO supplementation improved the antioxidant response, with no signs of intoxication, using a dose of 300 mg during the period of greatest oxidative stress.

## Introduction

Zinc (Zn) is a trace element abundant in biological systems, associated with over 300 enzymes in various biochemical, immunological, and cellular protection processes (Kabata-Pendias and Szteke 2015; VanValin et al. 2018). It is essential in cellular regulation mechanisms, gene transcription, and DNA replication, and also participates in the neutralization of reactive oxygen species (ROS) (Maywald et al. 2017; Robles et al. 2021).

In animal production, Zn is associated with strengthening the organism against pathogens and endoparasitic diseases (Goswami et al. 2005). It acts as an appetite modulator, promoting nutrient digestibility and animal growth (Cousins et al. 2006; Herdt and Hoff 2011). In ruminants, Zn contributes to the production of volatile fatty acids and the efficiency of energy generation (Swain et al. 2018), playing a role in energy metabolism and in maintaining the integrity of the ruminal epithelium, thus helping to prevent metabolic disorders (Mir et al. 2020).

Zn is supplied in small amounts in the diet, as the body does not have a specific organ for its storage (Herdt and Hoff 2011). In livestock production, the main sources used are inorganic forms, such as M-ZnO, a conventional supplement widely employed due to its low cost, biocompatibility, and low toxicity, in addition to its use in the pharmaceutical and cosmetic industries (Kolodziejczak-Radzimska and Jesionowski, 2014). However, M-ZnO shows limited interaction with the gastrointestinal tract, which reduces its bioavailability. In this context, the N-ZnO has stood out due to its smaller size and larger surface area, characteristics that increase interaction with cellular structures and consequently its biological efficacy (NSTC, 2004; Feng et al. 2009; Abdelnour et al. 2021).

N-ZnO represents a promising alternative for micromineral supplementation, increasing Zn utilization along the gastrointestinal tract of ruminants (Abdollahi et al. 2020; Swain et al. 2021), promoting effects on biochemical, hematological, and immunological parameters of animal physiology (Swain et al. 2021), as well as providing antioxidant protection (Yusuf et al. 2023), even when administered at lower doses than conventional forms (Swain et al. 2018). Therefore, it constitutes an efficient strategy in animal production systems (Hassan et al. 2020; Patra and Lalhriatpuii 2020; Swain et al. 2024).

Late pregnancy and early lactation, even in sheep farming, are phases that represent a critical moment in animal metabolism due to physiological changes associated with the simultaneous need to meet nutritional demands for proper fetal development and the onset of milk production (Nawito et al. 2016; Abuelo et al. 2019; Jouanne et al. 2021), making it a period that requires greater attention in animal production.

Therefore, N-ZnO may be an alternative to conventional Zn sources, such as M-ZnO, increasing the mineral’s bioavailability and optimizing the response of blood metabolic markers, which may result in better physiological adaptation to the transition period and improved productive performance of ewes. Thus, the present study aims to evaluate the effects of daily supplementation with 300 mg/animal/day of N-ZnO during the transition period, comprising late gestation through early lactation, in Santa Inês breed ewes.

## Material and methods

### Experimental procedures

The project was approved by the Ethics Committee on Animal Use of the Center for Nuclear Energy on Agriculture from University of São Paulo (CENA/USP 001/2021). The experiment was conducted at the Animal Nutrition Laboratory Animal Facility, located at the Center for Nuclear Energy in Agriculture (CENA), with geographic coordinates 22°23’31’’S, 47°38’57’’W and an altitude of 570 m, in Piracicaba, São Paulo, Brazil. Thirty-three Santa Inês ewes were used, with an average body weight of 43.3 ± 2.05 kg, in good health and reproductive condition, multiparous, and carrying single pregnancies confirmed by rectal ultrasonography. Capsules were previously prepared using a manual encapsulator and calibrated with a precision balance accurate to 0.0001 g. The experiment began at 60 days of gestation and was conducted until 60 days after parturition. The capsules were administered daily in the morning, before the feed was provided. The treatments were distributed as follows: control group (CON): capsules containing only ground grass hay (placebo) (*n* = 11); N-ZnO group: 300 mg of 40 nm N-ZnO (MKNano^®^, Mississauga, Canada) (*n* = 11); and microparticle zinc oxide group (M-ZnO) (Bauminas Agro, MG-Brazil): 300 mg of conventional-size M-ZnO (*n* = 11).

The zinc doses used in this experiment were based on previous studies, such as that of Mena et al. (2025), which support the use of doses up to 150 mg/animal/day in lambs naturally infected with gastrointestinal nematodes, due to the higher bioavailability of N-ZnO, promoting improvements in the immune system and growth performance without reaching toxic levels. In the present study, a dose of 300 mg/animal/day was chosen due to the importance of the transition period between pregnancy and lactation, characterized by increased oxidative stress and nutritional demands. Furthermore, at this stage, the immune system tends to receive secondary priority compared to growth and reproductive functions, especially in the presence of parasites (Coop and Kyriazakis 1999).

The diet was established according to the nutritional requirements recommended by the NRC (2007), considering the physiological stage of the ewes. Therefore, the concentrate was formulated with 70% ground corn, 15% soybean meal, and 15% cottonseed meal, on a dry matter basis. During gestation, 400 g/animal/day of concentrate were provided, with the amount adjusted to 600 g/animal/day during lactation. The feed was divided into two daily meals, offered in the morning and afternoon, maintaining the same proportions of ingredients.

During the gestational period, the ewes were kept during the daytime in paddocks of Aruana grass (*Megathyrsus maximus* cv. Aruana), with an average paddock area of 689.3 ± 38.65 m². In the late afternoon, the animals were gathered to receive the second portion of concentrate, remaining overnight with access to commercially sourced Tifton hay (*Cynodon* spp.). During the lactation period, the ewes were kept in a confinement system, with exclusive access to *Cynodon* spp. hay provided *ad libitum*. A Zn-free mineral salt with the following composition (g/kg of dry matter): CaHPO₄ (486.63), NaCl (264.80), MgO (123.56), S (72.63), FeSO₄ (35.06), MnSO₄ (11.38), CuSO₄ (2.31), KI (0.69), Na₂SeO₃ (1.50), and CoSO₄ (1.44) was provided in polyethylene troughs distributed among the experimental treatments. Both the mineral salt and the water were supplied ad libitum throughout the entire experimental period.

### Forage sampling and bromatological analysis

Bromatological analyses were carried out monthly for the forage. The concentrate ingredients and the hay, in turn, were sampled with each new batch, at 21-day intervals between collections. The analyses were conducted following AOAC (2000) procedures to determine dry matter, organic matter (method 934.01), crude protein (method 2001.11), ether extract (method 2003.05), and mineral matter (method 942.05). Meanwhile, neutral detergent fiber and acid detergent fiber analyses were performed according to the protocol described by Van Soest et al. (1991), with adaptations proposed by Mertens et al. (2002), using a TE-149 fiber analyzer (Tecnal^®^, Piracicaba, SP, Brazil), the composition is described in Table 1.

The zinc content in foods was determined in 200 mg samples, subjected to pre-digestion with 4 mL of HNO₃ (65%) for 12 h, followed by the addition of 1 mL of H₂O₂ and homogenization. Digestion was performed in a microwave (1800 W; 150–200 °C), and after dilution with ultrapure water, zinc was quantified by triple quadrupole inductively coupled plasma mass spectrometry (TQ-ICP-MS) (Agilent 8900^®^). Water samples, collected at different experimental sites and subsequently pooled, were acidified with HNO₃ and analyzed directly by TQ-ICP-MS at the Radioisotope Laboratory (LRI/CENA-USP). The values are described in Table 1.

**Table 1:** Bromatological composition of ingredients on a dry matter basis (g/kg DM) and zinc content (mg/kg DM)

Bromatological composition	Feeds			
	Forage	Hay	Concentrate	Water
Dry matter	217.13	940.08	883.34	
Organic matter	854.68	930.10	972.76	
Crude protein	146.56	65.71	256.19	
Neutral detergent fiber	643.74	776.10	172.00	
Acid detergent fiber	398.10	497.98	134.52	
Ether extract	31.43	10.30	30.86	
Ash	24.06	58.75	28.68	
Zinc	43.76	33.00	47.34	0.091

### Weighing and control of endoparasites

Body weight monitoring was carried out from the beginning of gestation until weaning, at biweekly intervals. During the same period, the presence of gastrointestinal nematodes was also monitored every two weeks. Fecal samples were collected directly from the rectum of each animal and processed using the modified McMaster technique, in which each nematode egg counted corresponded to 100 eggs per gram of feces (EPG) (Leland 1995; Ueno and Gonçalves 1998). To identify the predominant genera of gastrointestinal nematodes, pooled fecal samples by treatment were grouped and used to perform coprocultures, following the protocol described by Ueno and Gonçalves (1998).

Ewes presenting EPG counts ≥ 4,000 and/or hematocrit values ≤ 25% were treated according to the health protocol established for the flock. This protocol consisted of the intramuscular administration of 2 mL of iron dextran (Ferrodex^®^, J.A. Saúde Animal, Brazil) to correct anemia, and the oral administration of 5% levamisole hydrochloride (Ripercol^®^, 1 L; Zoetis, Campinas, São Paulo, Brazil), at a dose of 1 mL/10 kg of body weight, for the control of endoparasite infection, aiming to prevent possible adverse effects throughout the experimental period.

### Blood collection and processing

Blood was collected by puncture of the jugular vein using vacuum tubes with and without the anticoagulant EDTA. Collections were performed at 30 and 15 days prepartum (-30 and − 15), on the day of parturition (0), and during the postpartum period at 3, 7, 15, 30, 45, and 60 days (weaning). Hematological variables were analyzed by automated complete blood count using the Sysmex pocH-100iV Diff^®^ equipment. Differential leukocyte counts were performed by optical microscopy after staining blood smears with a rapid stain (Panótico^®^). After hematological analyses, a 2 mL aliquot of whole blood was stored at − 20 °C. The remaining samples were centrifuged at a relative centrifugal force of 480 × g to obtain plasma and serum, which were carefully separated and stored in a freezer at − 20 °C for later analyses. The remaining erythrocytes were subjected to hemolysis, diluted at a ratio of 1:6 in Triton X-100 solution (5 mL/L), and subsequently stored at − 80 °C.

### Zinc determination in blood

The Zn concentration in whole blood was determined following the protocol described by Meyer et al. (2018), with modifications proposed by Laur et al. (2020). The samples were subjected to acid digestion in polypropylene tubes. Subsequently, the tubes were heated in a water bath at 60 °C for 90 min in order to promote complete digestion of the samples. After heating, the samples were cooled to room temperature and ultrapure water was added. They were then centrifuged for 3 min at 560 × g. The Zn concentration was determined by TQ-ICP-MS, using the 8900^®^-model equipment (Agilent Technologies).

### Blood metabolite analysis

Plasma metabolite concentrations were determined using commercial kits (Labtest Diagnóstica^®^, Brazil) and analyzed with a spectrophotometer (JENWAY, model 7315). The analyses included: Glucose: enzymatic GOD-Trinder method, read at 505 nm; Urea: enzymatic UV method, read at 340 nm; and Creatinine: colorimetric method with alkaline picrate, read at 510 nm. In serum: Total proteins: biuret colorimetric method, read at 545 nm; Albumin: colorimetric method with bromocresol green, read at 630 nm; Aspartate aminotransferase (AST) and Alanine aminotransferase (ALT): UV-IFCC kinetic methods, read at 340 nm. Additionally, β-hydroxybutyrate levels were assessed using a drop of whole blood with the portable meter Optium Xceed^®^ (Abbott Laboratories) and specific reactive strips for β-ketone (FreeStyle/Optium™).

### Cortisol analysis

Cortisol analyses were performed in plasma using the radioimmunoassay (RIA) technique with a commercial kit (Beckman Coulter^®^). For each analysis, 50 µL of the sample was incubated at room temperature (18–25 °C) with 500 µL of I¹²⁵-labeled tracer, under constant agitation in a shaker at 400 rpm. Concentration readings were carried out using an automatic Gamma counter Wizard^®^ 2470 (PerkinElmer^®^).

### Cellular immune response

On day 15 postpartum, the cervical region of the animals was carefully cleaned and shaved, followed by marking a circle approximately 2 cm in diameter. In this area, a delayed-type hypersensitivity (DTH) test was performed using phytohemagglutinin (PHA) (Invitrogen^®^ 10576015) as a mitogenic agent, as described by Kegley and Spears (1995). Skin thickness was measured using a professional caliper. Then, 100 µL of PHA was administered intradermally on the right side of the circle, while 100 µL of phosphate-buffered saline (PBS) solution was administered on the left side as a control. Skin thickness measurements were taken immediately after application and again at 6, 24, and 48 h.

### Humoral immune response

To assess humoral immunity, on day 15 postpartum, the animals were challenged via intramuscular injection of 2 mg of ovalbumin (Sigma^®^ A5503), previously well mixed and diluted in 4 mL of a 1:1 (vol/vol) mixture of PBS and incomplete Freund’s adjuvant (IFA) (Pierce^®^ 77145). On day 20 after the injection, blood samples without EDTA were collected for serum separation and stored at -80 °C until analysis, with repeated sampling on the same day and again 20 days after the initial application.

Serum samples were subjected to an enzyme-linked immunosorbent assay (ELISA) for ovalbumin. Microplates were coated with ovalbumin (10 µg/ml, ovalbumin, Sigma REF: A5503-1G) for 1 h at 26 °C and blocked with 3% gelatin (Amresco, USA) in PBS (pH 7.2). Serum samples (1:20) were added in duplicate and incubated for 2 h. After washing, peroxidase-conjugated anti-sheep secondary antibody (1:10,000) (NB7195, Novus Biologicals, USA) was added for 1 h. The substrate was added, the reaction was stopped with 3 M sulfuric acid, and the reading was performed at 405 nm. Values were corrected by the blank and expressed as the mean of the duplicates.

### Immunoglobulin M (IgM)

IgM quantification in serum was performed by radial immunodiffusion, according to the method described by Mancini et al. (1965). The analyses were conducted on agarose plates containing sheep anti-IgM (Sigma^®^, SAB3700755). After sample application, the plates were incubated for 24 h in humid chambers to allow the formation of precipitation arcs. A previously established standard curve with bovine IgM (Sigma^®^, I8135) was performed for quantification. Serum IgM concentrations were expressed in mg/mL.

### Antioxidant analysis

SOD activity was determined in erythrocyte hemolysate using a commercial kit (Cayman Chemical, USA). The reaction involved a radical detector solution and xanthine oxidase, with incubation for 30 min at room temperature. One unit of SOD (U/mL) corresponds to the amount of enzyme capable of promoting 50% dismutation of the superoxide radical.

GPx activity was determined in erythrocyte hemolysate according to the protocol described by Wendel (1981). The samples were incubated in a reaction solution containing 48 mM phosphate buffer (pH 7.7), 0.38 mM EDTA, 0.95 mM sodium azide (catalase inhibitor), 1 mM reduced glutathione (GSH) (Sigma-Aldrich^®^, G4251-1G), 0.12 mM NADPH (Sigma-Aldrich^®^, 10107824001), 3.2 U glutathione reductase (Sigma-Aldrich^®^, G3664), 0.02 mM DL-dithiothreitol (DTT) (Sigma-Aldrich^®^, D0632), and 0.0007% hydrogen peroxide (H₂O₂) (Sigma-Aldrich^®^, 1072091000). The results were expressed in U/mL/Hb, where one unit of enzymatic activity corresponds to the amount of enzyme capable of catalyzing the oxidation of 1 µmol of reduced glutathione (GSH) to oxidized glutathione (GSSG) per minute, in the presence of H₂O₂, at 37 °C and pH 7.0.

Cat activity was determined in erythrocyte hemolysates according to the methodology described by Iwase et al. (2013). The reaction solution consisted of 100 µL of the sample (or standard), 100 µL of 1% Triton X-100, and 100 µL of 30% hydrogen peroxide (H₂O₂) (Sigma-Aldrich^®^, 1072091000), added to glass tubes. After incubation for 15 min at room temperature, the height of the formed oxygen (O₂) foam was measured using a digital caliper. The calibration curve was constructed using known units of catalase activity (Sigma-Aldrich^®^, C1345). One unit of catalase was defined as the amount of enzyme capable of decomposing 1 µmol of H₂O₂ per minute.

Total antioxidant status was evaluated in plasma samples using the ORAC assay (Oxygen Radical Absorbance Capacity), as described by Melo et al. (2015) and Moretti et al. (2019). For this purpose, samples were incubated with fluorescein and 2,2′-azobis(2-amidinopropane) dihydrochloride (AAPH) (Sigma-Aldrich^®^, ref. 440914). Quantification was based on a standard curve constructed with 6-hydroxy-2,5,7,8-tetramethylchroman-2-carboxylic acid (Trolox) (Sigma-Aldrich^®^, ref. 238813), and the results are expressed as mM of Trolox equivalents per mL. Fluorescence readings were performed using a BioTek Synergy HTX microplate reader, with excitation at 485 nm and emission at 528 nm.

### Statistical analysis

The data were analyzed using PROC GLIMMIX (SAS 9.4^®^) in a completely randomized design with repeated measures over time. Levene’s and Shapiro-Wilk tests were applied and, when necessary, a log10(x + 1) transformation was performed. Means were compared using Tukey’s test (*P* < 0.05). The treatment decision (EPG ≥ 4000) and iron supplementation were evaluated by mixed-effects logistic regression. Results are presented as means ± standard error, with graphs generated in SigmaPlot v.11.

## Results

**Fig. 1:**
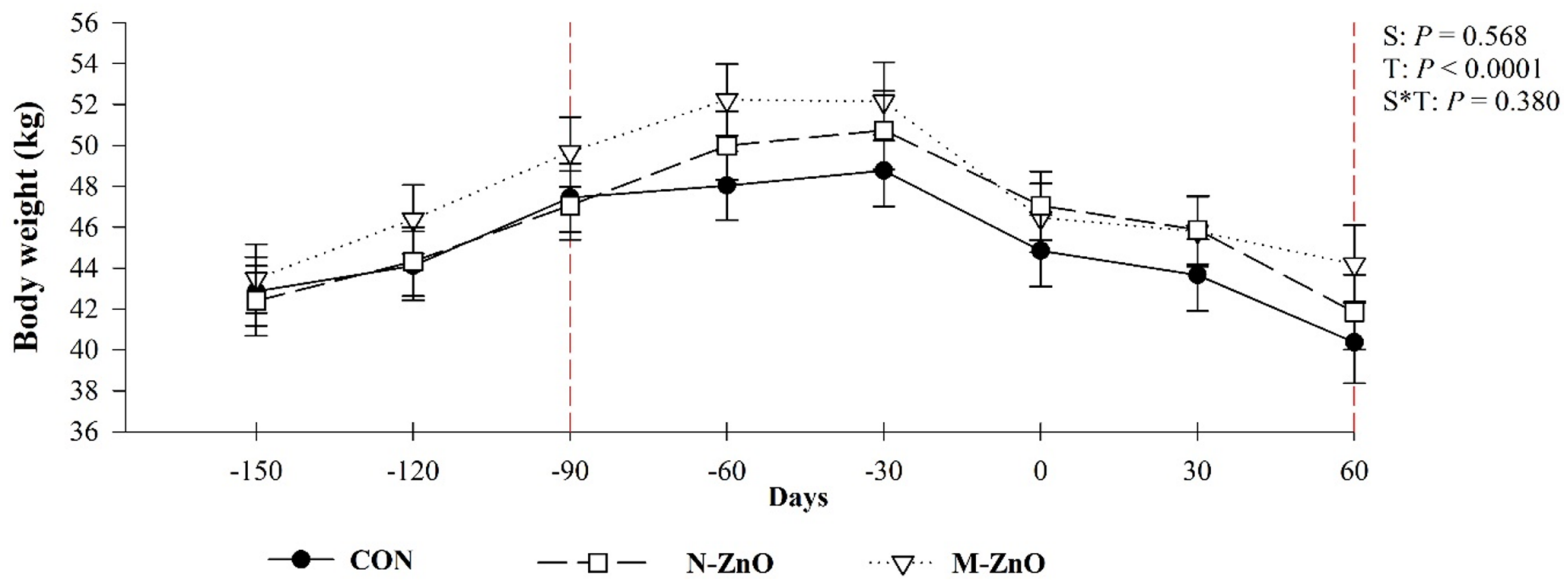
Mean ± standard error of body weight (BW) of ewes over time across different treatments. The points on the X-axis represent the days relative to parturition (Parturition = 0), with supplementation starting on day − 90 prepartum. S = supplementation effect; T = time effect; S*T = interaction between supplementation and time

**Fig. 2:**
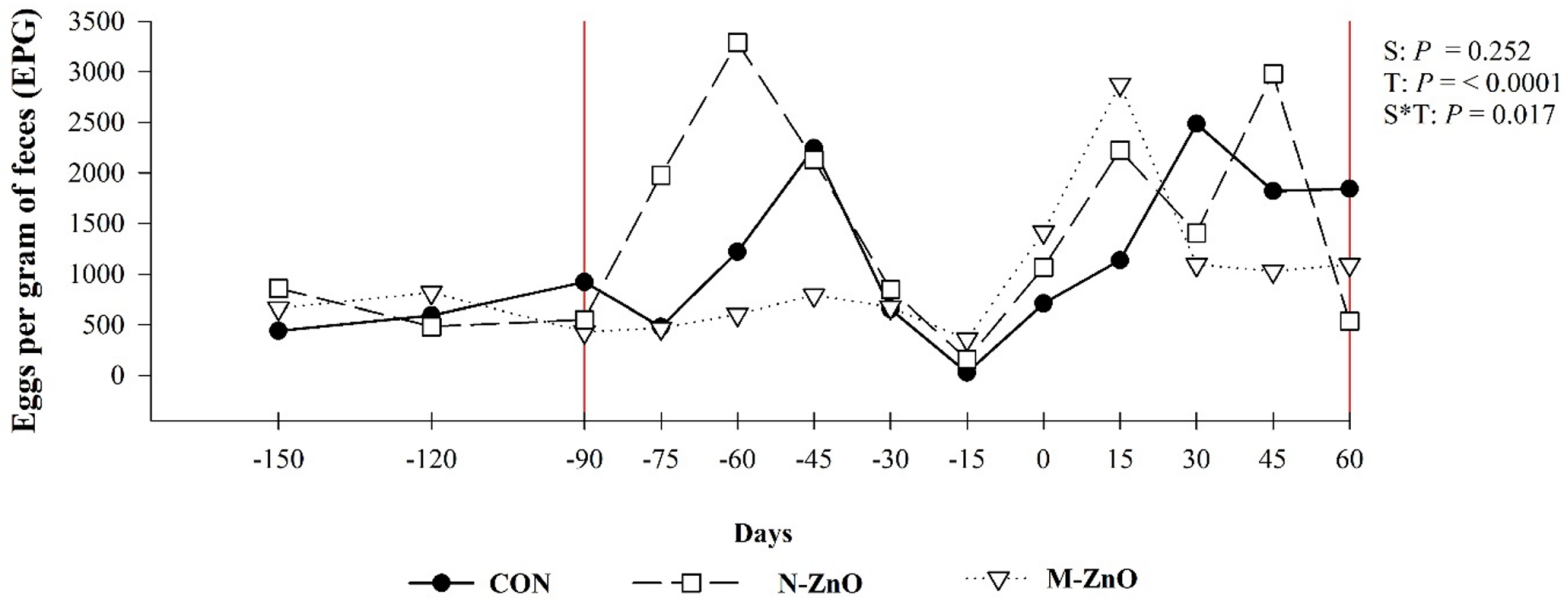
Mean of eggs per gram of feces (EPG) of ewes over time. The points on the X-axis represent the days relative to parturition (Parturition = 0), with supplementation starting on day − 90 prepartum. S = supplementation effect; T = time effect; S*T = interaction between supplementation and time

The condition of the sheep, assessed by body weight (BW) (Fig. 1) and FEC (Fig. 2) values, did not differ between the experimental groups (*P* > 0.05). However, a time effect was observed (*P* < 0.05), with a progressive increase in BW from the beginning of gestation (-150) to the end of gestation, followed by a reduction after parturition until weaning. EPG values increased during the pre- and postpartum periods, corresponding to physiologically critical phases (late gestation, parturition, and peak lactation). Due to the high EPG counts, it was necessary to perform antiparasitic treatment.

**Fig. 3:**
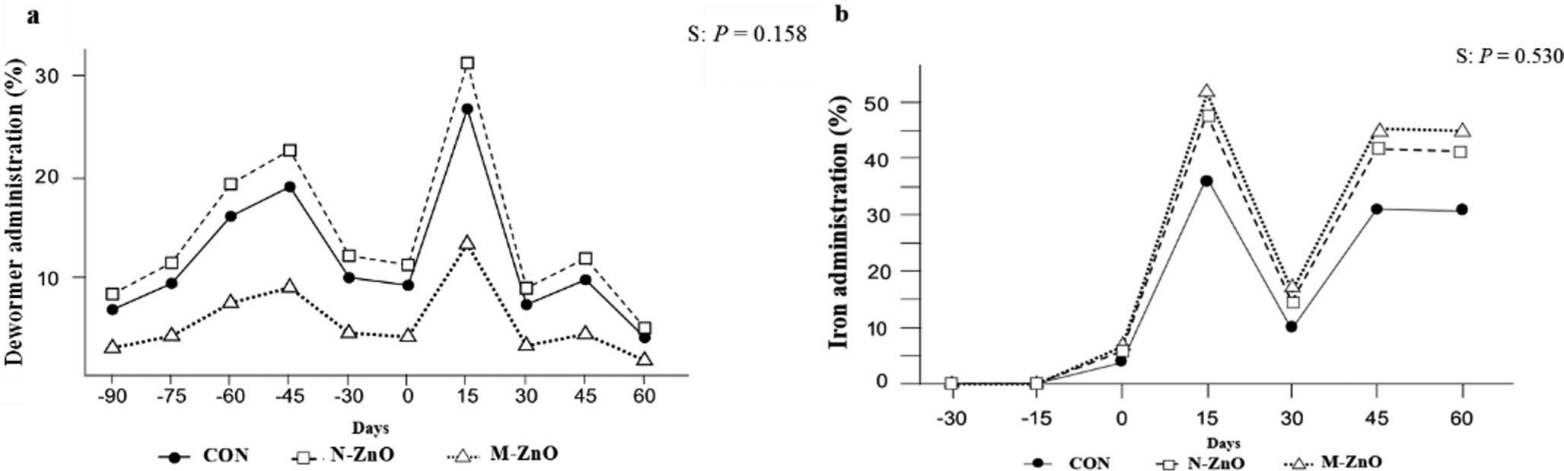
Proportion of animals receiving dewormer (**a**) and iron administration (**b**). The points on the X-axis represent the days relative to parturition (Parturition = 0). S = supplementation effect

Logistic analysis (Fig. 3) showed the proportion of animals treated over time, with no differences between treatments (*P* > 0.05). Anthelmintic treatments were mainly required at the end of gestation and during lactation due to the increase in gastrointestinal nematode burden, whereas iron administration was required only during lactation and was given independently of the anthelmintic treatment.

Coprocultures indicated that *Haemonchus spp.*, was the predominant genus throughout the entire experimental period, with frequencies above 80%. *Trichostrongylus* spp. was more prevalent at the beginning of the evaluation (1–49%), decreasing thereafter. *Cooperia* spp. was detected only sporadically. *Oesophagostomum* spp. showed moderate occurrence at specific times, especially in the CON and M-ZnO groups, reaching up to 27% and 25%, respectively, and reappearing in the final samplings.

**Fig. 4:**
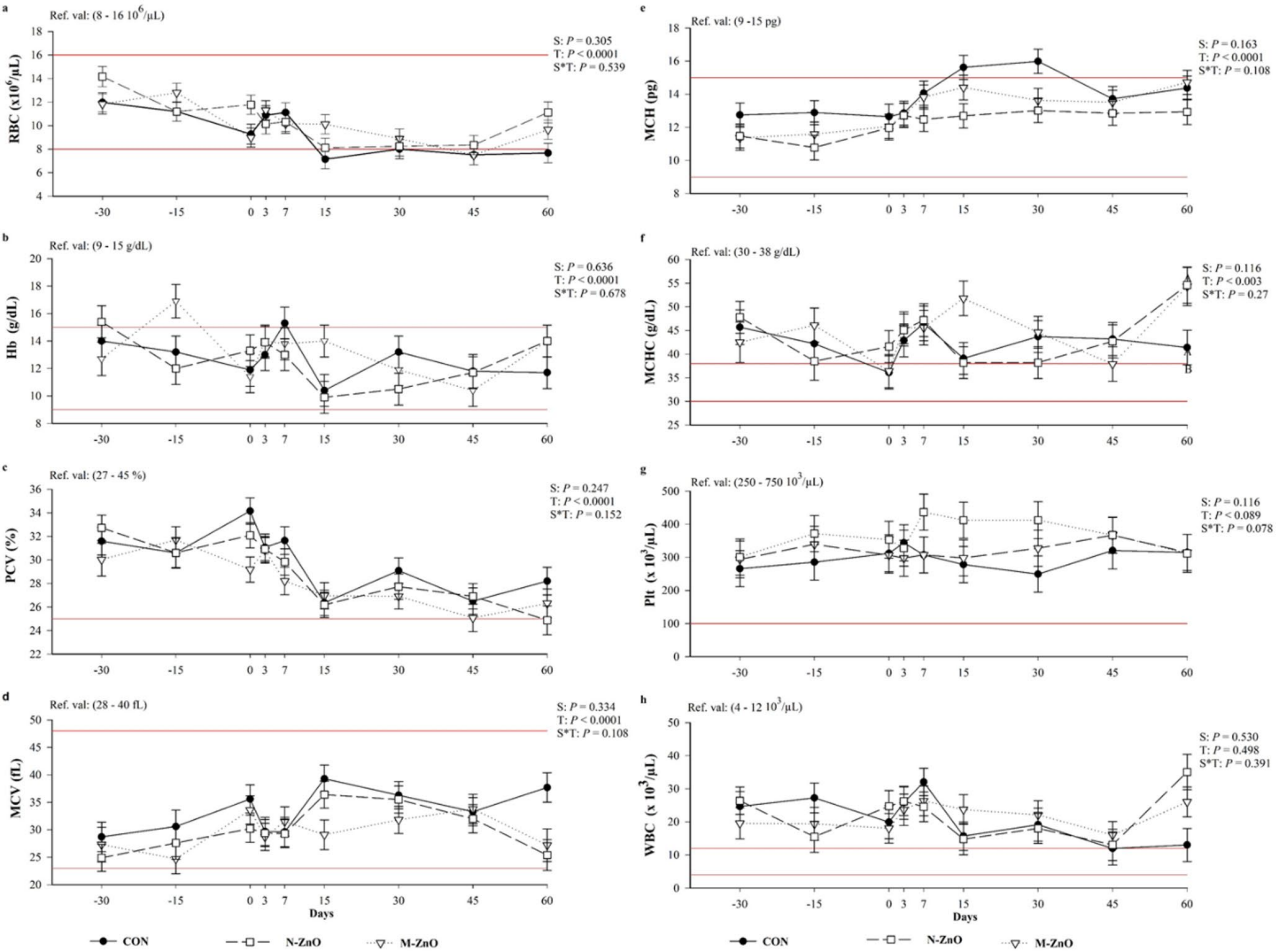
Mean ± standard error of hematological variables: (**a**) red blood cells (RBC), (**b**) hemoglobin (Hb), (**c**) hematocrit (Hct), (**d**) mean corpuscular volume (MCV), (**e**) mean corpuscular hemoglobin (MCH), (**f**) mean corpuscular hemoglobin concentration (MCHC), (**g**) platelets (Plt), and (**h**) white blood cells WBC. The points on the X-axis represent the days relative to parturition (Parturition = 0). Reference value (Ref. val) according to Kaneko et al. (2008): red horizontal line. S = supplementation effect; T = time effect; S*T = interaction between supplementation and time

**Fig. 5:**
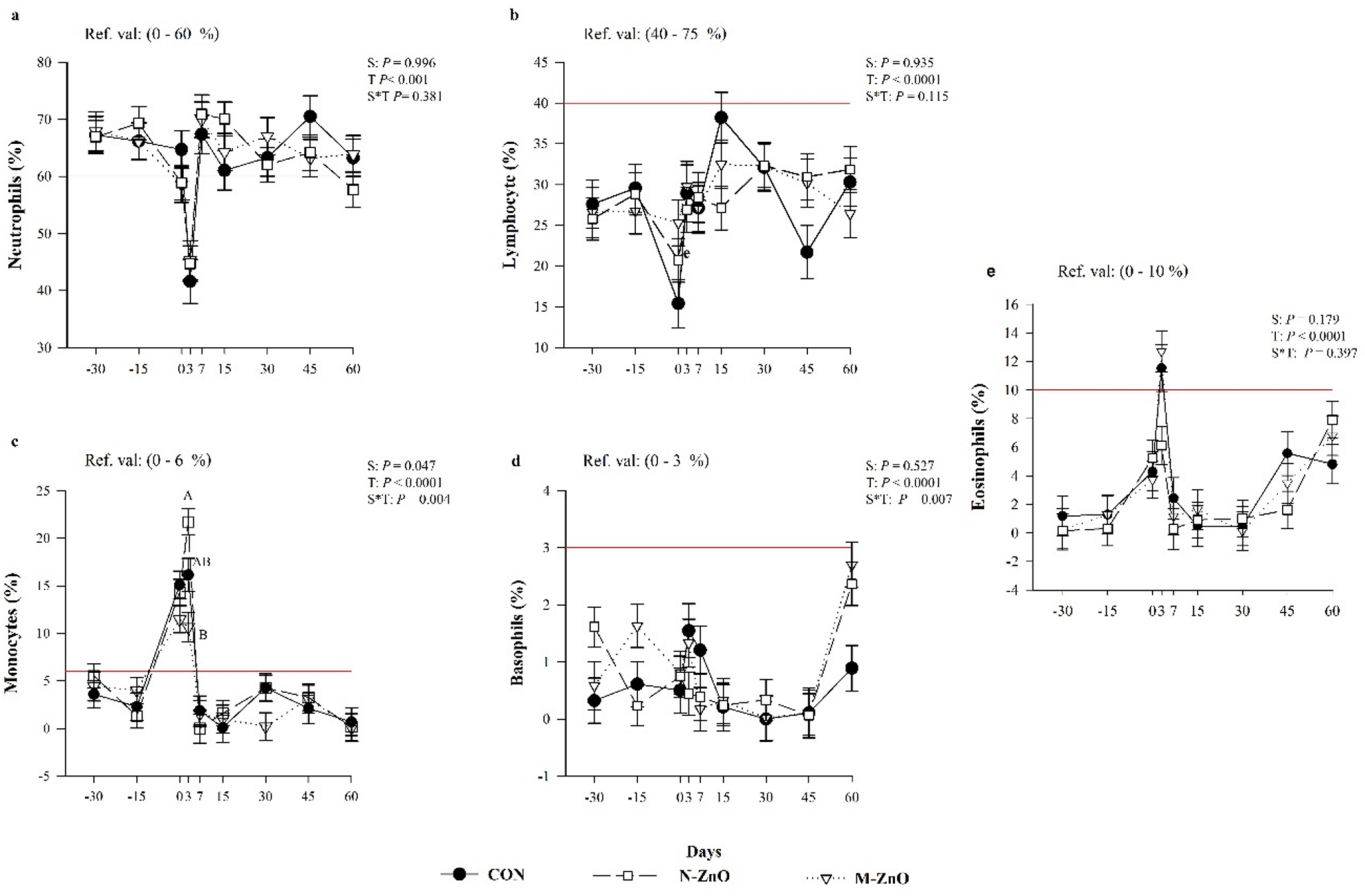
Mean ± standard error of leukocyte differential percentage: (**a**) neutrophils, (**b**) lymphocytes, (**c**) monocytes, (**d**) basophils, and (**e**) eosinophils. The points on the X-axis represent the days relative to parturition (Parturition = 0). Reference value (Ref. val) according to Kaneko et al. (2008): red horizontal line. S = supplementation effect; T = time effect; S*T = interaction between supplementation and time. Uppercase letters indicate differences between groups: A, B, C

For the hematological variables presented in Fig. 4, as well as the leukocyte differential distribution (neutrophils, lymphocytes, monocytes, eosinophils, and basophils) (Fig. 5), no differences were observed among the experimental groups (*P* > 0.05), except for monocytes, which showed higher values on the third day postpartum in the N-ZnO group.

Differences over time (*P* < 0.05) were observed in most hematological variables, except for platelet count, which remained within reference values throughout the experimental period. RBC, PCV, and Hb decreased after parturition, with partial recovery by day 60. In contrast, MCV, MCH, and MCHC increased progressively during the postpartum period. MCHC and total leukocyte counts remained above the reference ranges described by Kaneko et al. (2008).

In the variables of the leukocyte differential (Fig. 4), neutrophils decreased around day 3 after parturition, but remained above the reference values thereafter. Lymphocytes declined on days 0 and 3, with gradual postpartum recovery, although still below the reference range. Monocytes and eosinophils increased on days 0 and 3 and subsequently returned to reference values. Basophils were higher on day 60 but remained within normal physiological limits.

**Fig. 6:**
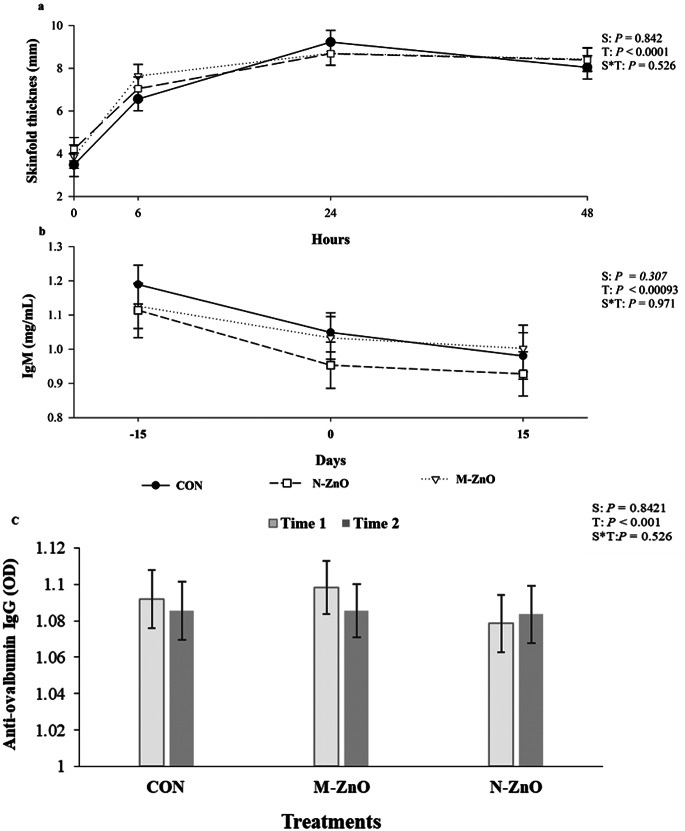
Mean ± standard error of (**a**) delayed-type hypersensitivity (DTH) response measured by skinfold thickness at different times after stimulation; (**b**) Immunoglobulin M (IgM) concentration; (**c**) anti-ovalbumin Immunoglobulin G (IgG) concentration measured by optical density (DO); time 1 (35 days postpartum), time 2 (55 days postpartum). S = supplementation effect; T = time effect; S*T = interaction between supplementation and time

The cellular immune response, assessed by skinfold thickness (Fig. 6a), did not differ among experimental groups (*P* > 0.05) but increased over time (*P* < 0.05). Serum IgM concentration (Fig. 6b) also showed no differences among treatments (*P* > 0.05), although it decreased progressively until day 15 postpartum. Similarly, specific anti-ovalbumin IgG levels (Fig. 6c) remained unchanged across treatments and experimental periods (*P* > 0.05).

**Fig. 7:**
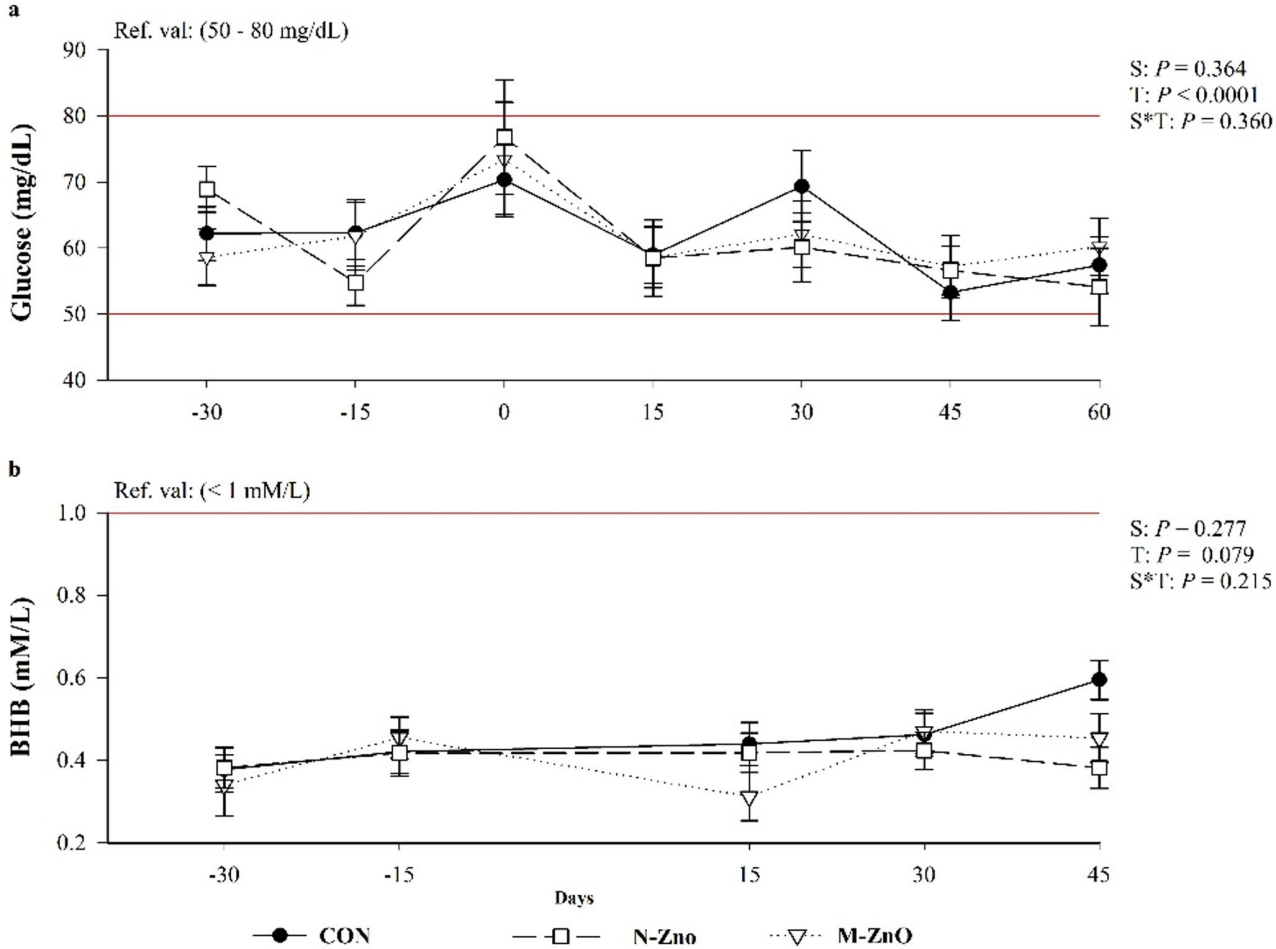
Mean ± standard error of the energy profile: (**a**) glucose and (**b**) β-hydroxybutyrate (BHB). The points on the X-axis represent the days relative to parturition (Parturition = 0). Reference value (Ref. val) according to Kaneko et al. (2008): red horizontal line. S = supplementation effect; T = time effect; S*T = interaction between supplementation and time

**Fig. 8:**
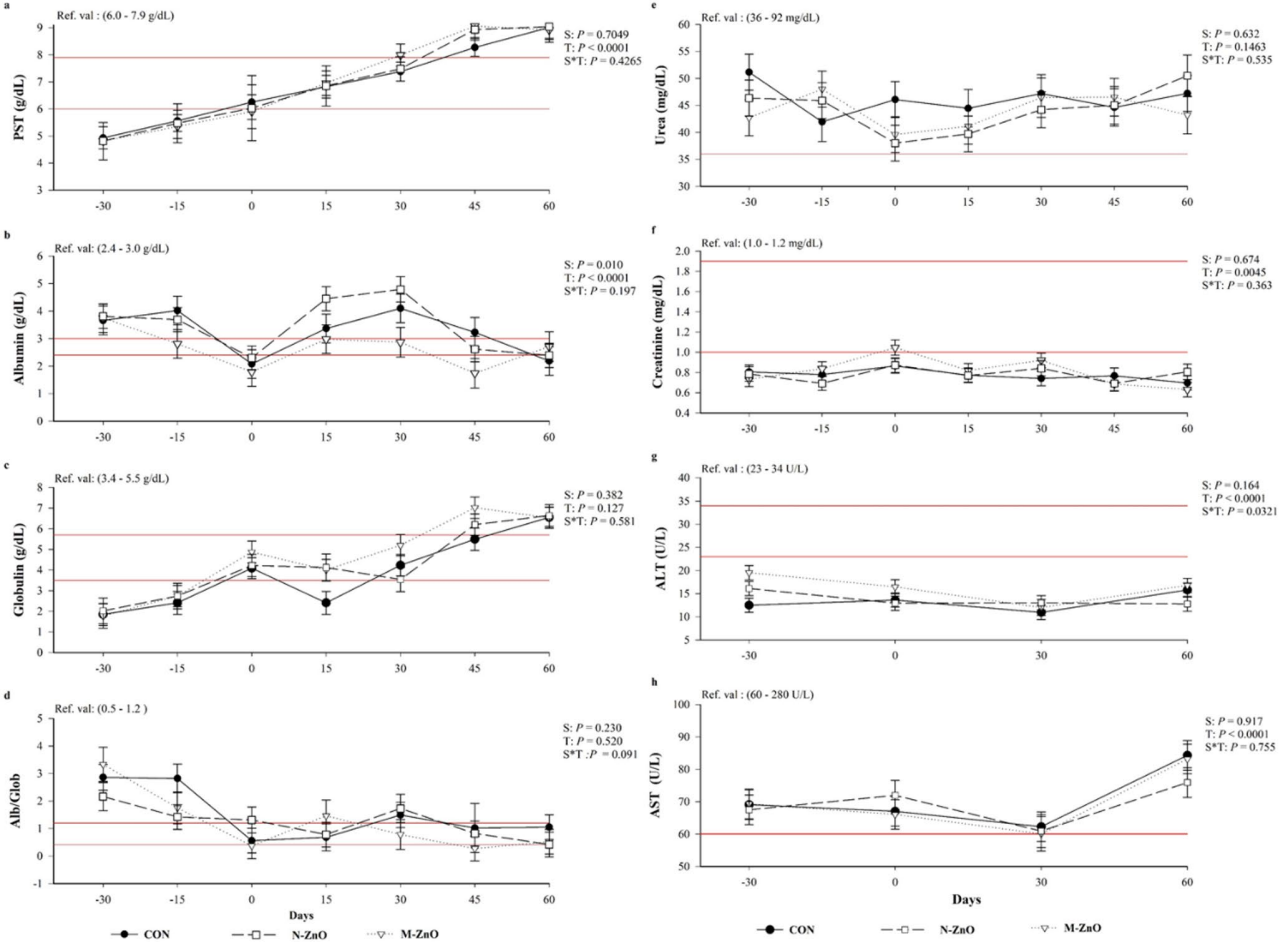
Mean ± standard error of the protein profile: (**a**) total serum proteins (TSP), (**b**) albumin, (**c**) globulin, (**d**) albumin/globulin ratio (Alb/Glob). Of liver and kidney function profile (**e**) urea, (**f**) creatinine, (**g**) alanine aminotransferase (ALT), and (**h**) aspartate aminotransferase (AST). The points on the X-axis represent the days relative to parturition (Parturition = 0). Reference value (Ref. val) according to Kaneko et al. (2008): red horizontal line. S = supplementation effect; T = time effect; S*T = interaction between supplementation and time

Blood metabolic variables (Figs. 7 and 8) showed no differences among treatments (*P* > 0.05), except for albumin (*P* < 0.05). The N-ZnO group presented a higher mean compared to the M-ZnO group, while the CON group showed intermediate values, without differing from the supplemented groups. There was a time effect (*P* < 0.05) for glucose, total serum proteins (TSP), albumin, urea, creatinine, aspartate aminotransferase (AST), and alanine aminotransferase (ALT). Glucose was higher on day 0 and decreased thereafter, remaining within the reference values. TSP and globulin increased gradually, while albumin decreased at parturition and at 45 and 60 days.

**Fig. 9:**
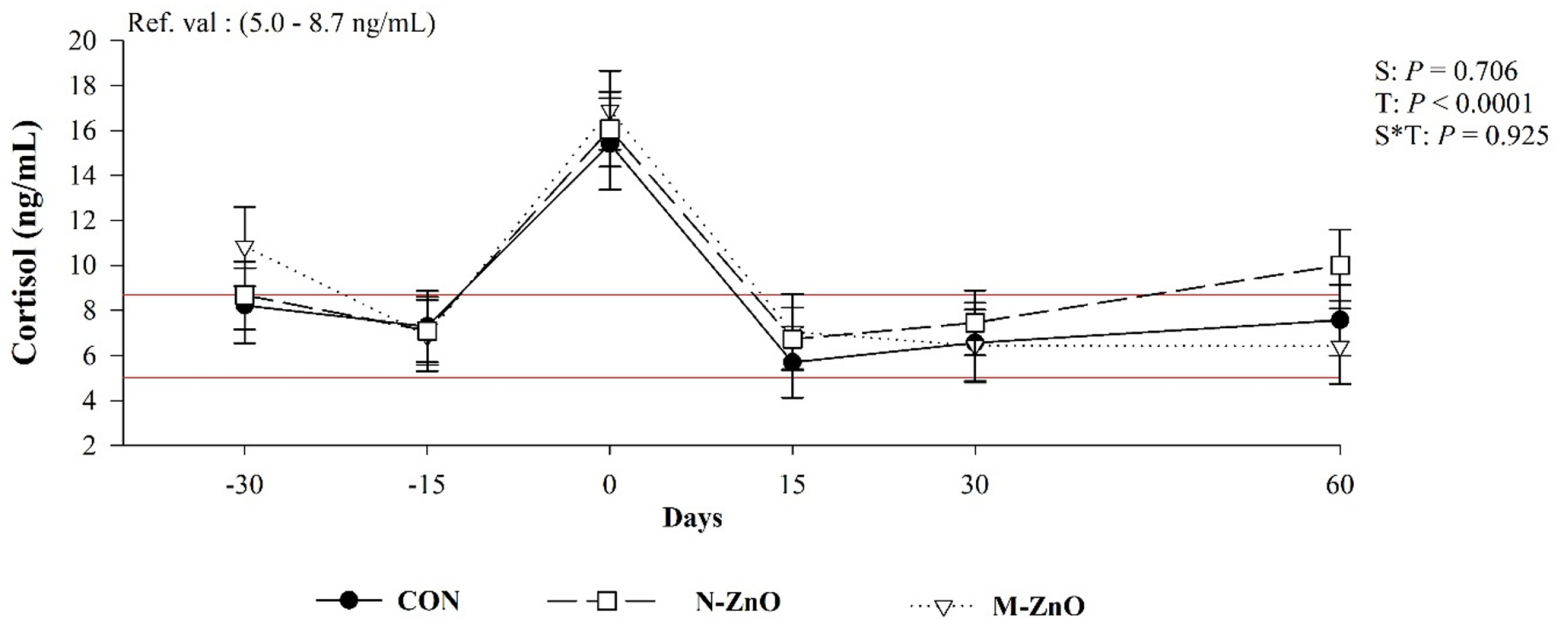
– Mean ± standard error of cortisol concentrations. The points on the X-axis represent the days relative to parturition (Parturition = 0). Reference value (Ref. val) according to Kaneko et al. (2008): red horizontal line. S = supplementation effect; T = time effect; S*T = interaction between supplementation and time

The evaluation of cortisol concentration (Fig. 9) showed no differences among treatments (*P* > 0.05), but changes were observed over time (*P* < 0.05), with a peak identified on day 0, associated with the birthing period.

**Fig. 10:**
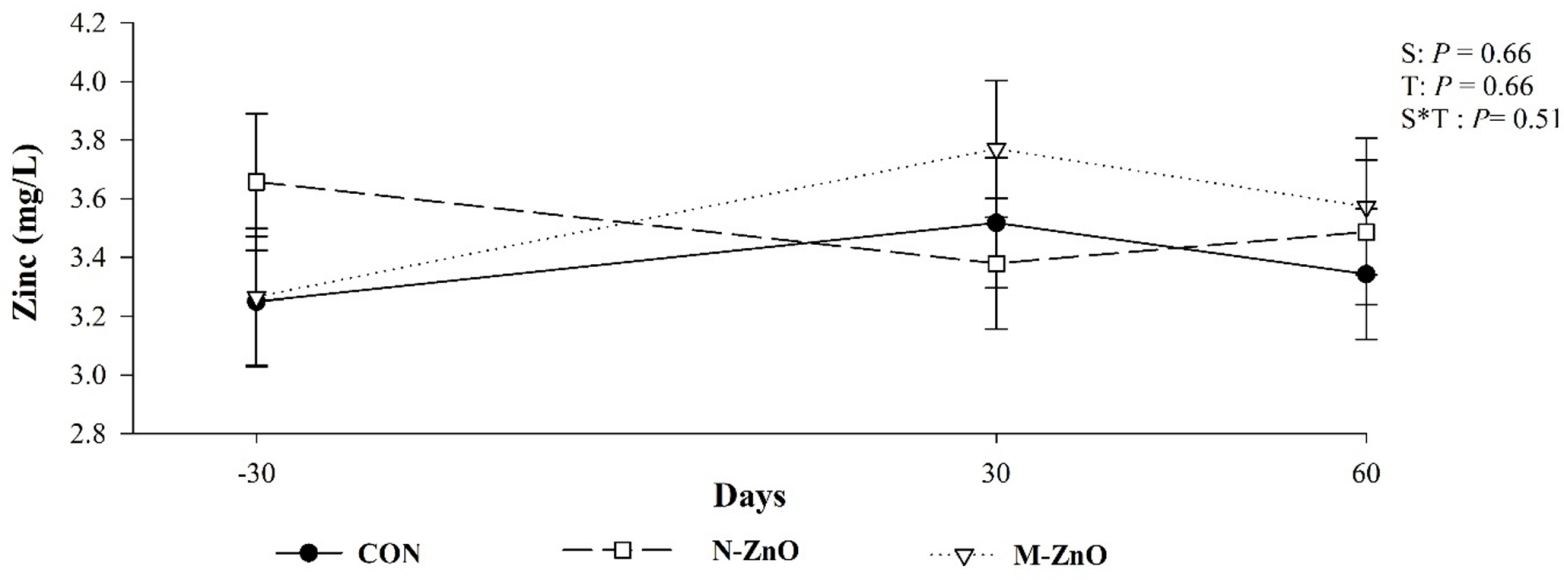
– Mean ± standard error of zinc concentration in whole blood. The points on the X-axis represent the days relative to parturition (Parturition = 0). S = supplementation effect; T = time effect; S*T = interaction between supplementation and time

In blood Zn concentration (Fig. 10), no significant differences were observed either among the experimental groups or over time (*P* > 0.05).

**Fig. 11:**
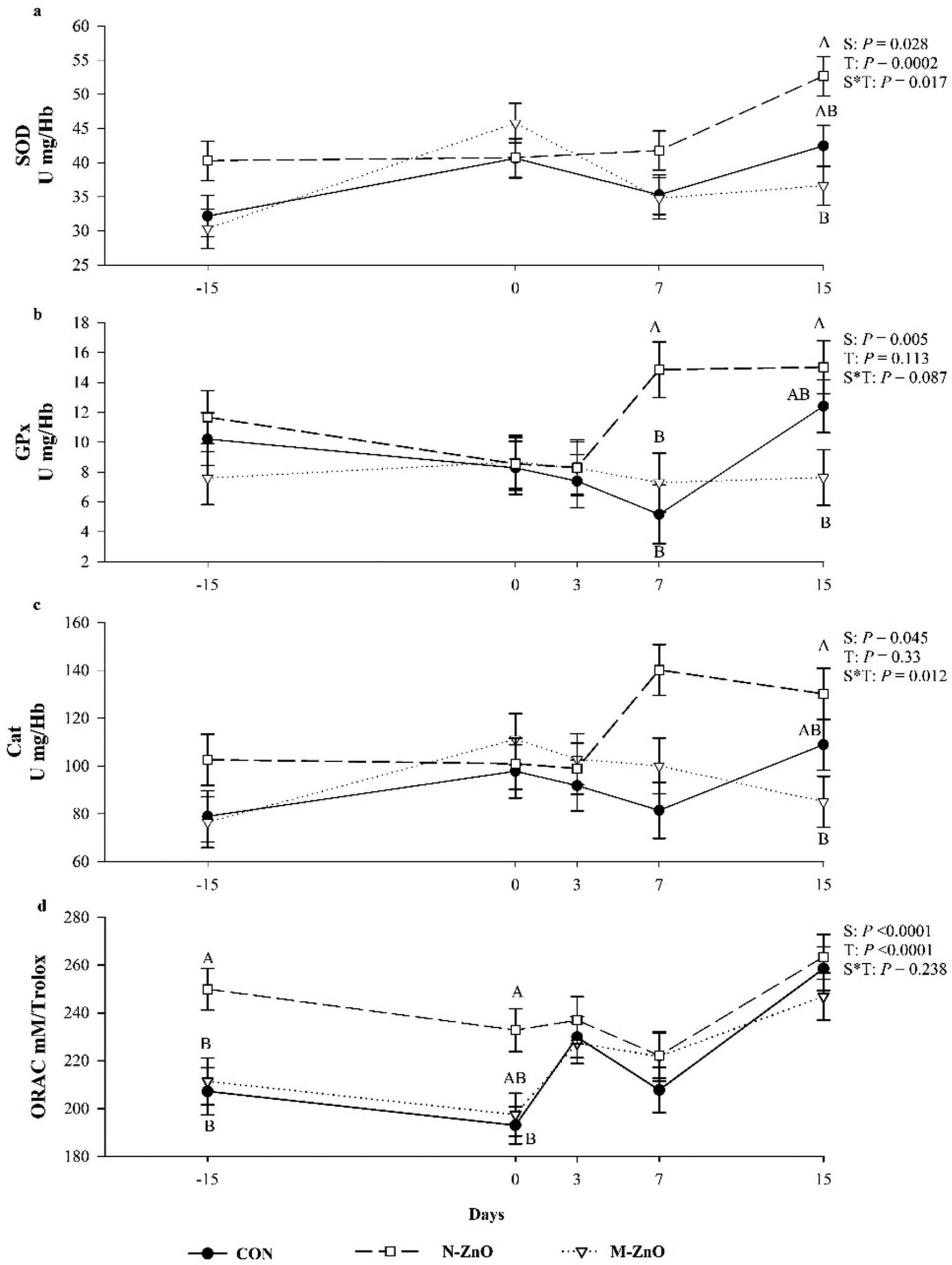
– Mean ± standard error of the antioxidant profile: (**a**) superoxide dismutase (SOD), (**b**) glutathione peroxidase (GPx), (**c**) catalase (Cat), and (**d**) total antioxidant capacity (ORAC). The points on the X-axis represent the days relative to parturition (Parturition = 0). S = supplementation effect; T = time effect; S*T = interaction between supplementation and time. Uppercase letters indicate differences between groups: A, B, C

The activity of antioxidant enzymes (SOD, GPx, and CAT) and antioxidant status (ORAC) (Fig. 11) showed a treatment effect (*P* < 0.05), with a time effect for SOD and ORAC. On day 15, the N-ZnO group presented higher SOD activity, while M-ZnO showed the lowest values and CON was intermediate. GPx activity was also higher in the N-ZnO group, standing out on day 7 postpartum; on day 15, the CON group differed from the supplemented groups.

Catalase (CAT) activity followed a pattern similar to that of SOD, with a greater response in the N-ZnO group on day 15 postpartum, differing from M-ZnO, but not from CON, which showed intermediate values. Regarding antioxidant capacity (ORAC), the N-ZnO group presented higher values than CON from day − 15 onward, without differing from M-ZnO. After parturition, no differences were observed among the groups, and on day 15 postpartum a marked increase in antioxidant capacity was observed in all groups.

## Discussion

The conditions of the ewes indicate that nutritional management, both in terms of the quality and quantity of the diet offered, was adequate to meet nutritional and energy requirements. Furthermore, considering that the basal Zn content in the diet ranged from 33 mg/kg of DM in the hay to 47.34 mg/kg of DM in the concentrate, together with 0.091 mg/kg in the drinking water, it may represent adequate Zn levels in the animals according to the NRC (2007) recommendations (30 mg/animal/day in the last third of gestation and 44 mg/animal/day in early lactation). Additionally, blood Zn concentrations were observed within the range considered normal (3.0–4.0 mg/L), values compatible with those reported for sheep (Ademi et al. 2017; Gatti et al. 2025) and goats (Kachuee et al. 2019). Corroborating what was reported by Mena et al. (2025) in lambs supplemented with 150 mg/day of N-ZnO compared with a non-supplemented control group, highlighting the regulatory capacity of the organism in the face of high intakes of this mineral in different physiological states, as discussed by Robles et al. (2021).

N-ZnO is often associated with potential toxic effects in different organisms; in this study, supplementation of 300 mg/day was established considering the physiological state of the ewes, aiming to observe the effect of daily supplementation. However, hepatic (AST and ALT) and renal (creatinine, urea) biochemical parameters remained within the reference values for the Santa Inês breed (Do Nascimento et al. 2015; Van Saun 2023), indicating the absence of adverse effects from high supplemental levels of ZnO, both in conventional size and nanoparticle form, during the transition period, corroborating the safety of N-ZnO use in animal production (Swain et al. 2016; Singh et al. 2018).

On the other hand, albumin, a protein involved in maintaining colloid osmotic pressure and the main transporter of nutrients in the blood (Jouanne et al. 2021), is especially important during periods characterized by greater mobilization and transport of nutrients due to physiological changes associated with the transition period (Park et al. 2010). According to Handing et al. (2016) and Mohamed et al. (2017), the albumin values observed in the N-ZnO group may indicate greater Zn bioavailability in the gastrointestinal tract, as well as adequate amino acid availability for hepatic protein synthesis, a mechanism that may favor the production and stability of serum proteins during lactation (Mira et al. 2023).

The presence of gastrointestinal nematodes in this study followed a typical pattern observed under grazing conditions in tropical regions. In this context, the adoption of a sanitary management plan during the experimental period was essential to keep the animals in good condition. However, this strategy may have contributed to the absence of beneficial effects from Zn supplementation observed in other studies, such as the increased resistance to *H. contortus* described by Baldissera et al. (2015) and the anthelmintic effects of N-ZnO against the same parasite reported by Esmaeilnejad et al. (2018).

In addition, hematological parameters were influenced, with only fluctuations observed that are physiologically expected during the peripartum period, both for cellular recruitment and uterine involution, including a reduction in neutrophil count around day 3 postpartum, as reported in cows (Hoedemaker, 1992). Moreover, cortisol levels during the peripartum period, according to Fonteque et al. (2013), may modulate lymphocyte populations. While the modulation found in monocytes in the N-ZnO group still lacks direct evidence of Zn-mediated activation (Kesler, 2024), it is considered that the greater bioavailability may have contributed to monocyte activity in inflammation resolution and tissue regeneration processes, as described by Olechnowicz et al. (2018). However, it is noteworthy that no significant differences were observed in the humoral or cellular immune response under daily N-ZnO supplementation.

In the present study, supplementation with N-ZnO positively influenced the activity of antioxidant enzymes during the transition period. An increase in SOD activity was observed, a zinc-dependent metalloenzyme essential for protecting cell membranes against oxidative damage (Fadayifar et al. 2012; Marreiro et al. 2017). In addition, increased activities of Cat and GPx were verified, enzymes responsible for neutralizing ROS, which act synergistically in maintaining redox balance (Burton and Jauniaux 2011), demonstrating how Zn bioavailability provides indirect activation of antioxidant enzymes (Oteiza 2012).

Therefore, the results indicate that supplementation with N-ZnO promoted an efficient antioxidant response compared to the conventional form (Hosseini-Vardanjani, 2020; Kumar et al. 2021; Pandey et al. 2023). Thus, N-ZnO proved to be a promising alternative for controlling oxidative stress in ewes during the transition period, mediated by the enzymes SOD, CAT, and GPx. Furthermore, it acts as an immunological promoter of monocytes during the peripartum period. It is noteworthy that no signs of intoxication were observed with daily supplementation of 300 mg of N-ZnO (40 nm), reinforcing its viability as a micromineral supplement.

## Data Availability

The datasets generated and analyzed during the current study are not publicly available due to the possibility of generating additional data that may complement this study, but are available from the corresponding author on reasonable request.
